# Examining dependencies among different time scales in episodic memory – an experience sampling study

**DOI:** 10.3389/fpsyg.2023.1277741

**Published:** 2024-01-11

**Authors:** Hyungwook Yim, Paul M. Garrett, Megan Baker, Jaehyuk Cha, Vishnu Sreekumar, Simon J. Dennis

**Affiliations:** ^1^Department of Cognitive Sciences, Hanyang University, Seoul, Republic of Korea; ^2^School of Psychological Sciences, The University of Melbourne, Melbourne, VIC, Australia; ^3^School of Psychology, The University of Newcastle, Callaghan, NSW, Australia; ^4^Department of Computer Science, Hanyang University, Seoul, Republic of Korea; ^5^Cognitive Science Lab, International Institute of Information Technology, Hyderabad, India

**Keywords:** time scale dependency, independence of scales, experience sampling, episodic memory, autobiographical memory, memory for when

## Abstract

We re-examined whether different time scales such as week, day of week, and hour of day are independently used during memory retrieval as has been previously argued (i.e., independence of scales). To overcome the limitations of previous studies, we used experience sampling technology to obtain test stimuli that have higher ecological validity. We also used pointwise mutual information to directly calculate the degree of dependency between time scales in a formal way. Participants were provided with a smartphone and were asked to wear it around their neck for two weeks, which was equipped with an app that automatically collected time, images, GPS, audio and accelerometry. After a one-week retention interval, participants were presented with an image that was captured during their data collection phase, and were tested on their memory of when the event happened (i.e., week, day of week, and hour). We find that, in contrast to previous arguments, memories of different time scales were not retrieved independently. Moreover, through rendering recurrence plots of the images that the participants collected, we provide evidence the dependency may have originated from the repetitive events that the participants encountered in their daily life.

## 1 Introduction

**Figure 1:**
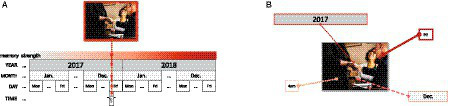
Theories that explain how people retrieve different time scale information of an event. **(A)** Strength hypothesis, and **(B)** Reconstructive hypothesis. Image source: https://pxhere.com/en/photo/1540055 (CC0 Public Domain).

When trying to remember when a past event happened, people are able to retrieve time information from different scales such as the year, month, day of month, and hour of the event (e.g., Friedman and Wilkins, 1985). How are people able to remember different time scales of an event and how are memories of different time scales represented? Friedman and Wilkins (1985) examined a couple of hypotheses. One reasonable hypothesis was that time information is estimated by the strength of the memory that decays over time.^1^ In this case, one is estimating a single point back in time based on the memory-strength continuum (see Figure 1A). Since a single point back in time is associated with different hierarchical time scales, even though people may not try to intentionally or explicitly access different time scales, the strength hypothesis (e.g., Hinrichs, 1970) predicts that different time scales naturally become interdependent. Moreover, since coarser time scales have a wider coverage on the continuum, the strength-based view predicts that if a finer time scale (e.g., hour) is correctly remembered, a coarser time scale (e.g., year) will likely be remembered. Consequently, a directional dependence exists in remembering time scales, where the probability of correctly remembering a coarser time scale is affected by the probability of correctly remembering a finer time scale.

**Figure 2:**
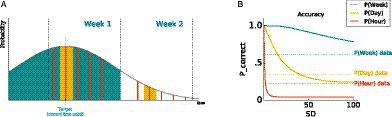
Simulation results from a formal strength model being applied to the current study. **(A)** An example of the model when the correct time point (target) was 10 am, Tuesday, Week 1. Probability correct of each time scale could be derived from the area under the curve – P(week) shaded in green, P(day) shaded in yellow, and P(hour) shaded in orange, where the area for the wider range (e.g., green) includes the narrower range (e.g., yellow, orange). **(B)** Accuracy of each time scale as a function of the noise distribution (SD), where accuracy data from the current study is also plotted in dotted lines.

To illustrate the dependency, we present a simulation using a toy model of the strength hypothesis as follows (see Figure 2). Note that this is a simplified version of the model to illustrate the overall phenomenon and does not include many detailed factors that can influence the pattern (e.g., boundary effect; Huttenlocher et al., 1992). Suppose one is trying to remember an event during a two-week vacation, and the true event happened on Week 1, Tuesday 10 am. Following the strength hypothesis, there will be a specific strength attached to this time point, and we will assume that there will be some noise, which follows a normal distribution centered on the target time point (see Figure 2A). Then the probability correct of the week scale (i.e., week 1) can be estimated by calculating the area under the curve where the memory strength is smaller than the border of week-1 and week-2 (shaded in green in Figure 2A). Probability correct for the day and hour scale can also be calculated in the same fashion. However, for the day scale there will be two Tuesdays one for each week (shaded in yellow), and for the hour scale there will be 10 points for 10 am, one for each weekday (shaded in orange). Moreover, as shown in Figure 2A, the area under the curve for the week scale is the largest, which results in the highest accuracy, followed by the day scale, and hour scale. Following this method, Figure 2B shows the probability correct for the three scales, where we took the average of all possible target time points in the study. Then we examined whether the noise of the signal would affect the results by changing the standard deviation of the normal distribution, which is presented through the *x*-axis (SD). Regardless of the degree of noise in the signal, the model always predicts that the coarser time scale (i.e., week scale) will be more accurately retrieved than the finer time scale (i.e., hour scale) – the green line (i.e., week scale) is always on the top while the orange line (i.e., hour scale) is always on the bottom of the accuracy plot shown in Figure 2B.

On the other hand, Friedman and Wilkins (1985) provided evidence that time scales are not linked to each other as the strength hypothesis proposes, but rather, retrieval cues for each time scale exist (reconstructive hypothesis; see Figure 1B). In their study, participants were presented with popular news events (e.g., John F. Kennedy’s assassination), and were asked about when the events happened on different time scales (e.g., year, month, day of month, day of week, and hour). Results showed that in some cases remembering a finer time scale was more accurate than remembering a coarser time scale (i.e., scale effects). Scale effects support the idea that people could use different cues to retrieve different time scales of the event rather than only relying on the overall memory strength of an event (Friedman, 1996). Similar results have been reported using different materials. For example, Friedman (1987) asked participants about when a local earthquake happened, Huttenlocher et al. (1992) asked participants, who previously responded to a phone survey, the day of week and time of the phone survey, and Larsen and Thompson (1995) asked when events in participants’ diaries happened. Although Friedman and Wilkins (1985) originally provided evidence for the scale effects to support the reconstructive hypothesis, the results have been interpreted as evidence also for independent time scales, which predicts that correctly remembering one time scale is unaffected by remembering another time scale (e.g., Friedman, 1996; Neath and Surprenant, 2002).

However, it is hard to conclude that time scales are independent from these results for two main reasons. First, it is possible that the materials used in previous studies are not fully representative of our day to day life events. Historical and media events (e.g., John F. Kennedy’s assassination) may have less self-relevance than our day to day events, or may be more salient than the typical events that occur on a daily basis (e.g., local earthquake). Diary studies have the issue of selection bias, where more salient events are more likely to be recorded by the participants than regular events (Sreekumar, 2015). An alternative way to examine the nature of time scale representation with better ecological validity is using passive experience sampling techniques. Experience sampling has the advantage of collecting each participant’s day to day events automatically without selection-bias, and by utilizing modern smartphones, various modalities may be easily recorded such as time, images, sounds, GPS, and accelerometry. Previous memory studies using experience sampling techniques have been successful in showing interesting findings about human memory in real life ranging from the kinds of cues people use to remember when an event happened, to how time and space are represented in the brain (e.g., Sreekumar et al., 2014; Nielson et al., 2015; Chow and Rissman, 2017; Dennis et al., 2017; Sreekumar et al., 2018).

Second, previous studies have not used a formal measure of dependency. Although the results from these studies (e.g., scale effects) serve as a counter-example against the strength hypothesis, they are not sufficient to support the claim that time scales are independent. A proper measure of dependency, such as pointwise mutual information (PMI; Fano, 1961) between time scales, is required. PMI is a way to formally measure the association between two events. Conceptually, PMI is the ratio between how two events occur together (i.e., P(A, B)), and our expectation of their appearance assuming the two events are independent (i.e., P(A)ꞏP(B)). The method has been frequently used in statistics, information theory, and natural language processing to measure the dependency among two events.

Therefore, in the current study we used experience sampling techniques to examine whether memories of different time scales are independently used and represented (i.e., independence of scales), and whether scale effects are present in everyday life. We also utilize a formal measure of dependency (i.e., PMI) to examine the magnitude of dependencies among different time scales. In the experiment, participants collected their day to day life events for 2 weeks using a smartphone which automatically collected various kinds of information including images of their surroundings and the time of when these images where taken. Then, participants were presented with images that they had collected and were asked what week, day of week, and hour of day the event depicted by the image happened. Additionally, we asked how confident the participants were in making each judgment.

## 2 Experiment

### 2.1 Methods

#### 2.1.1 Participants

Nineteen adults^2^ participated in the study (10 females, *M* = 26.47 yrs., *SD* = 6.30 yrs). Participants were recruited from flyers posted around campus and were paid AU$100 for their time and effort. The research was approved by The University of Newcastle Human Research Ethics Committee.

#### 2.1.2 Materials

The images used in each participant’s experiment were selected from each participant’s data, which was accumulated during the data collection period. To exclude images that were too blurry for the participant to identify or that contained no information (e.g., black image that may have been taken by mistakenly blocking the camera lens), we first by filtered out images that had entropy values below 17.0 or variation of the Laplacian (Pech-Pacheco et al., 2000) below 7.0. Then one image for each one-hour slot was selected based on how different the image was compared to other images in other time slots. The difference between images was calculated by the Euclidean distance of each image’s *gist* representation (Oliva and Torralba, 2001), where the image with the highest minimum-distance was selected for a given hour slot. For example, assume there are three images (e.g., A, B, C) in a given hour slot. We calculate the distance (i.e., Euclidean distance of *gist* representations) between A and all other images outside of the given hour slot (e.g., X, Y, Z) and take the minimum value among them as a distance measure for image A (i.e., minimum-distance). We repeat this process for all images in the given time slot (i.e., images B and C). Then we pick the image that has the highest minimum-distance measure among the three in order to choose the image that is the most distinct from images of other hour slots. The method was used to automatically select an image that was distinct for a given hour bin, and which was not similar across other time bins. The method aids in decreasing the ambiguity when the participants are deciding when the image was taken. Since a different number of images were collected by each participant, the number of images used at test were different across participants (*M* = 67.58, *SD* = 27.17, *range* = 22–122).

#### 2.1.3 Procedure

**Figure 3:**
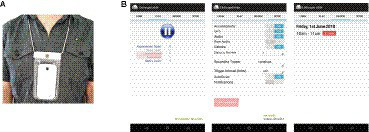
Apparatus used in the study. **(A)** Participants wore a smartphone around their neck during the data collection phase, **(B)** the layout of the Unforgettable app which was used for data collection. In order to ensure participant’s privacy, participants were able to turn on/off the whole app. (image on the left), or the recording of a specific sensor (image in the center), and were also able to delete events that were already recorded (image on the right). Adapted with permission from Unforgettable Technologies Pty Ltd.

There was a two-week data collection phase followed by a one-hour test phase, which was separated by approximately 7 days. The data collection phase always started on a Monday and ended on the Friday of the following week. During the data collection phase, participants were provided with a smartphone by the experimenter and were told to wear it around their neck during the weekdays when they were awake, as much as possible (see Figure 3A). The phone was equipped with the ‘Unforgettable’ app. (Unforgettable Technologies, 2017; Dennis et al., 2019), which collected image, time, audio (i.e., obfuscated information using mel-frequency cepstrum coefficients), GPS, accelerometer and orientation information every 5 min or when a movement was sensed by the phone (see Figure 3B for the layout of the app.). Participants had full control over the app. and could turn off the app. Anytime they needed privacy. The stored data was automatically sent to a remote server when the phone detected WiFi and was charged above 90%, which usually happened once per day when users charged the phone overnight.

**Figure 4:**
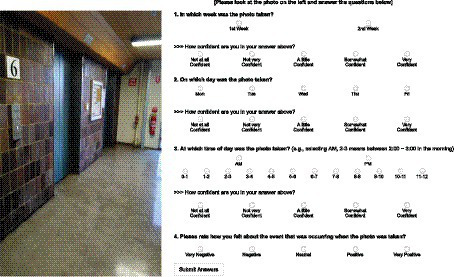
An example layout of a test trial that was administered online.

Seven days after the data collection phase (i.e., on the third Friday), participants were asked to login to an online webpage for the test phase. Participants were randomly presented with a selection of their images collected during the data collection phase. The images were presented one at a time on the left side of the screen with related questions on the right side (see Figure 4). Participants were asked in which week, day, and hour the event captured in the image happened, and were asked to make a confidence rating on a five-point scale for each response. The valence of the event was also elicited using a five-point scale. The number of test trials differed based on the number of images that were collected by each participant during the data collection phase (see Materials). The valence data is irrelevant to the current investigation and will be reported elsewhere.

In addition to the current task, a study-test memory task using the collected images was administered on the third Monday (i.e., approximately 4 days before the current test phase). Participants were presented with the images one at a time and had to remember the images, and after a delay were given a recognition memory task. The task was irrelevant to the current investigation in that participants did not make judgments or receive feedback about the time information of the images. The results of this task will be reported elsewhere.

#### 2.1.4 Description of calculation

##### 2.1.4.1 Deviation expected by chance (DEC)

We used error scores to examine the degree of accuracy following Friedman and Wilkins (1985). Error scores were calculated by taking the shortest distance between the participant’s response and the actual time, and then dividing the distance by the deviation expected by chance (DEC). DEC is the deviation that could be expected by random guessing, where it was 0.5 (= {0 + 1}/2) for Week, 1.2 (= {0 + 2·(1 + 2)}/5) for Day, and 3.23 (= {0 + 2·(1 + 2 + … + 6)}/13) for Hour, considering 13 h of data collection per day. Moreover, the shortest distance was defined by the difference in possible responses, and not by the physical distance between the participant’s response and the actual time. For example, if the correct answer was Friday for a day question and the participant responded as Monday, the shortest distance to the correct answer is 1 as data was not collected on the weekends. Since the DEC for day is 1.2 the error score is 0.83 (= 1/1.2).

##### 2.1.4.2 Pointwise mutual information (PMI)

To formally evaluate independence between different time scales, we used pointwise mutual information (PMI) as in Equation 1:


(1) $$PMI\left(A;B\right)=\log_{2}\left(\frac{P\left(A,B\right)}{P\left(A\right)\cdot P\left(B\right)}\right)$$

where, *P* (*A, B*) is the probability of correctly recalling both time scale A and B (e.g., week and day) of an event whereas *P* (*A*) and *P* (*B*) are the probabilities of correctly retrieving time scale A (e.g., week) and B (e.g., day) respectively. For example, if the probability of getting the week correct is 0.6, getting the day correct is 0.34, and getting the week and day correct is 0.23, PMI(week; day) = log_2_[0.23/(0.6·0.34)] = 0.17. PMI ranges from−∞ to min[−log_2_P(A), −log_2_P(B)], where a PMI of zero indicates that the two events are independent, whereas a value above or below zero indicates that the events are dependent.

## 3 Results

The pooled group data was analyzed with bootstrapping methods (Efron and Tibshirani, 1997) unless stated otherwise, as the number of trials varied by subject in that each subject’s data had a different level of reliability. The pooled group data was re-sampled by subject 1,000,000 times with replacement, and an empirical value of *p* was calculated for statistical inference, which is denoted by *p_empirical_*. The main analyses conducted on the subject-level are presented in the Supplementary material, where the results show a similar pattern as the current analyses but with more noise.

We first examined the accuracy for each time scale using a one-sample t-test against chance level. Although the chance level for *P* (*hour*) would be 1/24, most participants did not collect data for 24 h. The average of the maximum hour that participants collected data per day was 13.05 h (*SD* = 3.03, *range* = 9–21), and we used 1/13 as the chance level for *P* (*hour*).^3^ Results show that performance for all time scales were above chance (see Table 1), which indicates that participants were capable of recalling when an event happened in different time scales with reasonable precision. Participants also showed above chance performance in correctly remembering the exact week, day, and hour information of an event, *P* (*week, day, hour*) = 0.065 (*SD_bs_* = 0.011), chance-level = 0.008 (= 1/2 × 1/5 × 1/13), *p_empirical_* < 0.001. The error score for the day scale was the largest (*M* = 0.83, *SD*_bs_ = 0.04) followed by the hour (*M* = 0.79, *SD*_bs_ = 0.05) and week (*M* = 0.79, *SD*_bs_ = 0.04) error score, but the differences were only numerical (*p_empirical_* s > 0.05).

**Table 1:** Accuracy for each time scale with mean accuracy (M), standard deviation of the bootstrapped samples (SD_bs_), chance-level for each time scale, and Holm-Bonferroni corrected (HBC) empirical value of *p* against each chance-level derived from bootstrapping.

	*M*	*SD_bs_*	Chance-level	*p*-value
*P* (*week*)	0.61	0.022	0.50 (=1/2)	< 0.001
*P* (*day*)	0.34	0.029	0.20 (=1/5)	< 0.001
*P* (*hour*)	0.22	0.016	0.077 (=1/13)	< 0.001

**Figure 5:**
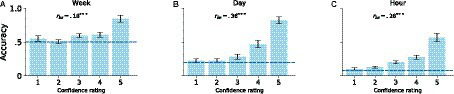
Accuracy by confidence rating for **(A)** week, **(B)** day, and **(C)** hour. Values on the *x*-axis represent confidence rating scores from ‘Not at all confident’ (1) to ‘Very confident’ (5). Dotted lines represent chance level for each time scale, error bars represent the standard deviation of the bootstrapped samples. Point biserial correlations (rbs) are presented for each time scale, where ∗∗∗ represents Holm-Bonferroni corrected empirical *p* < 0.001. Error bars represent ±1 standard deviation of the boostrapped samples.

Confidence ratings for the day scale (*M* = 1.56, *SD*_bs_ = 0.18) was lower than the hour (*M* = 1.82, *SD*_bs_ = 0.17, *p_empirical_* = 0.027) and week scale (*M* = 1.82, *SD*_bs_ = 0.21, *p_empirical_* < 0.001) using a randomization test with re-sampling by subject 1,000,000 times with replacement. The relationships between accuracy and response confidence at each time scale were also examined by calculating point bi-serial correlation coefficients (*rpb*; see Figure 5). *rpb* for the week (0.18), day (0.36), and hour scales (0.28) all showed significant correlations (*p_empirical_* s < 0.001; testing null-hypothesis as zero) replicating previous studies that show positive correlations between confidence and accuracy performance (e.g., Roediger and DeSoto, 2014).

The results from the error scores did not supported the fact that memory strength is the main source for retrieving memory for when, and support scale effects since there was no difference in accuracy between the time scale, and a tendency for the finer scale (i.e., hour) showing a better performance than the coarser scale (i.e., day). However, as discussed previously, the results do not provide direct evidence for the independence of time scales, and require a formal measure of independence such as point wise mutual information (PMI).

Table 2 shows PMIs calculated for different time scale pairs with value of *p*s from a one-sample *t*-test against zero. Results showed that all pairs were statistically different from zero (*p_empirical_* < 0.05). Although previous studies (e.g., Friedman and Wilkins, 1985) have posited that patterns in their data supported independence of time scales, utilizing a formal measure (i.e., PMI), the current results indicate that there are dependencies between the time scales.

**Table 2:** Pointwise mutual information (PMI) between different time scales with mean PMI (M), standard deviation of the bootstrapped samples (SD_bs_), and Bonferroni-Holm corrected empirical value of *p* against zero from bootstrapping.

	*M*	*SD_bs_*	*p*-value
*PMI*(*week*; *day*)	0.169	0.050	< 0.001
*PMI*(*week*; *hour*)	0.124	0.052	0.010
*PMI*(*day*; *hour*)	0.361	0.103	0.001

Results from the behavioral data support the idea that each time scale can be retrieved using its own retrieval cue (i.e., scale effects) but, at the same time, there are dependencies among the time scales. Thus, the time scales are not linked as the strength hypothesis assumes, but dependency still exists to a certain degree. One possible explanation for time scales being dependent is that cues for different time scales are correlated due to repeating schedules in everyday life.

A way to examine repeating events is by using recurrence plots (see Marwan et al., 2007, for a review). Recurrence plots are heat-maps of a distance matrix that allow one to examine the repeating patterns visually, and have been used in previous studies to identify repeating visual context (e.g., Sreekumar et al., 2014). To create recurrence plots, we followed the method of Sreekumar et al. (2014) by first converting images from RGB to HSV space, where the values were quantized into 192 colors (i.e., 12 hue, 4 saturation, and 4 lightness levels) for computational efficiency. Color correlograms were then calculated for each image (Huang et al., 1997). A color correlogram is a three dimensional table that describes the probability of finding one color (*Ci*) given another color (*Cj*) at a certain pixel distance (*k*). The color correlogram has been successful in distinguishing different contexts, as rated by people in previous studies (see Sreekumar et al., 2014, for comparing different image representations). For the current study, the summed color correlogram of *k* = {1, 3, 5, 7} was used as in Sreekumar et al. (2014). Then the distance matrix was constructed using the Euclidean distance of the color correlogram of each image.

**Figure 6:**
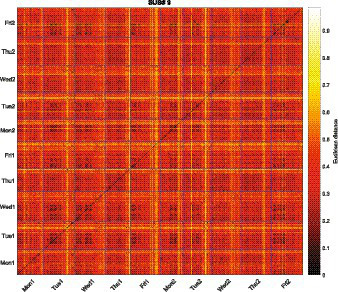
Recurrence plot for subject 9 using color correlogram image representation. Each intersection represents the Euclidean distance between the two corresponding images taken from the 1st Monday (MON1) to the 2nd Friday (FRI2). Blue lines represent the border of each day.

Figure 6 shows the recurrence plot for subject 9. Each point in the plot represents the distance between two images’ color correlogram ordered from the first Monday (Mon1) to the last Friday (Fri2), where the distance is color coded from black to white. For example, the diagonal from the bottom-left to the upper-right represents the distance between the identical images, and therefore shows all zero distances colored in black. In the plot, darker colors, which indicate similar visual context, can be identified around the diagonal of the first Tuesday (Tue1) and Wednesday (Wed1). These dark colors show that a context with similar visual representations is continuing for a period of time. For example, a class could be continuing for a period of time. Images taken during that time would be similar. Importantly, the dark patches could be identified on the off-diagonal as well. When looking at the column for the first Tuesday (Tue1), dark patches notably reappear at the intersection of Mon1, Wed1, Fri1, Mon2, Tue2, and Fri2. The recurring dark patches imply that a visual context similar to that of the first Tuesday is repeating on other days (e.g., the participant regularly attending class in a classroom). Formal measures show also support for the recurring patterns (Determinism, 0.4946, *p_empirical_* < 0.001; Average Diagonal Length, 3.1063, *p_empirical_* < 0.001; Divergence, 0.0039, *p_empirical_* < 0.001; Zbilut and Webber, 1992; Webber and Zbilut, 1994; see Supplementary materials for detailed description of the values and calculations). The recurrence plot provides evidence of events being repeated for subject 9 (see Supplementary material for similar patterns in all of the subjects’ recurrence plots), supporting the argument of different time-scale cues becoming more associated through repeating events.

## 4 Discussion

The current study examined whether memories of different time scales are independently represented. To overcome the shortcomings of the previous studies, we used experience sampling techniques to obtain a better representation of everyday life, and utilized PMI as a formal measure for independence. We find evidence that although each time scale is directly accessible (i.e., existence of the scale effects), different time scales are not independently represented as has been previously argued (i.e., PMI greater than zero for all time scale pairs).

Most importantly, evidence for dependencies among different time scales is an interesting and novel finding. Previous arguments that time scales are independent (e.g., Friedman, 1996; Neath and Surprenant, 2002) have been based on studies that show scale effects (e.g., Friedman and Wilkins, 1985; Friedman, 1987; Huttenlocher et al., 1992; Larsen and Thompson, 1995). However, the existence of the scale effects serves no more than a counter-example to falsify a pure strength hypothesis, which assumes that all time scales are perfectly dependent to each another (see Figures 1A, 2). The key contribution of the current study is instead using a formal (and direct) measure of dependency (i.e., PMI) to evaluate dependencies among time scales.

The current study also shows evidence for scale effects as the error score and confidence rating for the hour scale (i.e., finer scale) showed better performance than that of the day scale (i.e., coarser scale). The scale effects, which is argued by Friedman, imply that the dependency among time scales are not rooted in the simple strength hypothesis. The necessity of an additional or alternative mechanism to the strength-based mechanism is also shown in the formal version of the strength hypothesis that we introduced in the introduction (see Figure 2). Figure 2B shows probability correct for each time scale as we change the noise level in the model (i.e., SD; standard deviation of the noise distribution), and the dotted lines presents data from the current study. The model predicts almost perfect accuracy for week (green line) when the noise is small, and some degree of noise should be assumed to predict the accuracy level of the current study (i.e., 0.61). However, as we increase the noise level, accuracy for the hour scale (orange line) rapidly declines below the accuracy of the current study (i.e., 0.22). A similar pattern is shown for the accuracy of the day scale. The discrepancy between the model prediction and the actual data implies that a simple strength-based process proposed by Friedman is not enough to explain how different time scales are used and represented, and there are additional (or alternative) processes that aid the retrieval of a finer scale such as the hour or day scale. As suggested by Friedman and Wilkins (1985), a reconstructive hypothesis, or more specifically a location-based process (Friedman, 1996), could predict better retrieval accuracy for the finer scales. The location-based process, compared to the distance-based process that is mainly based on memory strength, assumes that there are cues associated with time scale information that enables one to “reconstruct” the time information (e.g., estimating the time of a local earthquake as 11:50 am based on the fact that the earthquake happened right before lunch time; Friedman, 1987). Therefore, the time scale that has a stronger cue associated with it will show better retrieval. However, an important point that is less discussed in these theories is that time scale dependency could be predicted when the cues are dependent. The recurrence plot from the current study shown in Figure 6 highly supports this idea. Considering that most of the participants were university students who have a fixed schedule, many of the events they experience may repeat, and different time scales in these events would be correlated, providing opportunities for two time scales cues to be repetitively encoded together (e.g., I have a Cognitive Psychology class on Mondays 3 pm). As the cues become more associated, retrieval of the time scales that are linked to these cues become more dependent. For example, the fact that the highest PMI is between the day and hour scale (i.e., 0.36) would reflect the fact that the participants, who were mostly university students, have more dependent cues for the hour and day scales through their academic timetables.

The notion that different time scales and cues are interdependent aligns with theories of autobiographical memory (e.g., Kolodner, 1983; Barsalou, 1988; Conway and Pleydell-Pearce, 2000), which propose that when we experience an event, we comprehend the event by retrieving both generic knowledge relevant to that event and specific, related prior events. For example, Barsalou (1988) described an autobiographical free recall experiment where participants were asked to describe the events they experienced in the prior summer in the order that they came to mind. Participants primarily described generic event types (e.g., several occasions of playing tennis) followed by specific events (e.g., a short event such as a picnic) and extended events (e.g., a job that extends across days, interrupted by evenings spent with family). Similar results were obtained in another experiment where they explicitly intervened to instruct participants to only describe specific events. Barsalou concluded that retrieving extended and generic event types was an important part of accessing information about a target period of one’s life and constructed a theory of autobiographical memory which was motivated by three findings: (1) the importance of chronologically organized extended events in free-recall verbal protocols, (2) other types of organization, such as by activity, people, and location, and (3) the prevalence of summarized (over multiple occurrences) event types in free-recall protocols. Of these, Barsalou identifies extended-event hierarchical timelines as central to providing people with a way of *telling time in autobiographical memory*. Barsalou’s theory (also see Conway and Pleydell-Pearce, 2000 for a similar view), along with the use of the location-based process, will also produce scale dependence. For example, a student-participant during a semester would not only have specific memories about each class she took, but also have built a hierarchical experience structure about their class schedule (e.g., Cognitive psychology class on Mondays at 3 pm). If the participant was asked to estimate the time of an event that was related to her Cognitive psychology class (e.g., meeting a friend right before the class), this information about the class would be used as a cue to retrieve the hour information (e.g., sometime before 3 pm since it was before the class). Moreover, since the cues are interlinked in the hierarchical structure, other time-scales will be more likely to be retrieved (e.g., it would be Monday since it was before the Cognitive psychology class I have on Mondays, etc.).

Another contribution of the current study is in the use of experience sampling methods to provide a way to capture better samples of our daily life. Regarding the current study, it would not have been possible using previous methods (e.g., using news events) to capture the repetitive nature of our daily life, and test each event that was captured (i.e., showing images as a query at test). As discussed earlier, it is highly possible that dependency among time scales stems from the repetitive events that participants encountered. This is not to say that samples from previous studies (e.g., Friedman and Wilkins, 1985) are invalid. Since previous studies did not formally measure dependency, it is possible that events that do not repeat and have a longer retention interval (e.g., asking when John F. Kennedy’s assassination was) may have dependency among time scales, and it would be a matter of future investigation. However, what experience sampling, which automatically logs one’s daily events, provides is a more uniform sample that covers both repetitive and non-repetitive events, and is a more ecologically valid sample of the memories of everyday life.

Although the current results support that people use information of different time scales interdependently when accessing ‘memory for when,’ we do not claim that this is the only mechanism to access ‘memory for when.’ For example, Friedman (1996) additionally proposed that people can retrieve when an event happened using the order (i.e., relative time) information between the events. This mechanism is closely related to the Source Monitoring Framework (Johnson et al., 1993), where it is argued that people infer when an event happened using various information that includes the strength of the memory, semantic details, and affective information. It would be valuable to consider different mechanisms in an integrated way for future studies. We also do not claim that the current results will apply to distant memories as we only examined memories within a month range. It is possible that more distant memories will be accessed through a distance-based process more frequently than a location-based process as specific schedules may not be accessible. Therefore, an important future study would be to examine the independence of time scales with more distant memories. Finally, testing all time scales at once may increase the interdependence across the time scales. Testing a single time scale at a time may be a useful future study to conduct.

## Data availability statement

The raw behavioral data supporting the conclusions of this article will be made available by the authors, without undue reservation.

## Ethics statement

The studies involving humans were approved by The University of Newcastle (Australia) Human Research Ethics Committee. The studies were conducted in accordance with the local legislation and institutional requirements. The participants provided their written informed consent to participate in this study.

## Author contributions

HY: Conceptualization, Data curation, Formal analysis, Funding acquisition, Investigation, Methodology, Project administration, Validation, Visualization, Writing – original draft, Writing – review & editing. PG: Data curation, Investigation, Methodology, Project administration, Software, Writing – review & editing. MB: Conceptualization, Data curation, Project administration, Writing – original draft. JC: Funding acquisition, Writing – review & editing. VS: Investigation, Validation, Writing – review & editing. SD: Conceptualization, Formal analysis, Funding acquisition, Investigation, Resources, Supervision, Validation, Writing – review & editing.

## Footnotes

^1^We acknowledge that time is not the only factor that determines memory strength, and external and mental factors can influence the strength of the memory. Here, we only consider time as our focus of interest.

^2^The number of participants were decided based on previous studies that used a similar method (Sreekumar et al., 2014; Nielson et al., 2015).

^3^Note that for the accuracy on the hour scale, we additionally conducted the *t*-test using individual chance-levels, and accuracy was still above chance-level (see Supplementary material).
