# Supplementary material for: Examining dependencies among different time scales in episodic memory – an experience sampling study
Source: Front Psychol. 2024 Jan 11;14:1277741. doi: 10.3389/fpsyg.2023.1277741 (PMC10808733; doi:10.3389/fpsyg.2023.1277741)
Supplement: Supplementary file 1 [file Data_Sheet_1.PDF]

Supplementary Materials

1 Individual recurrence plots

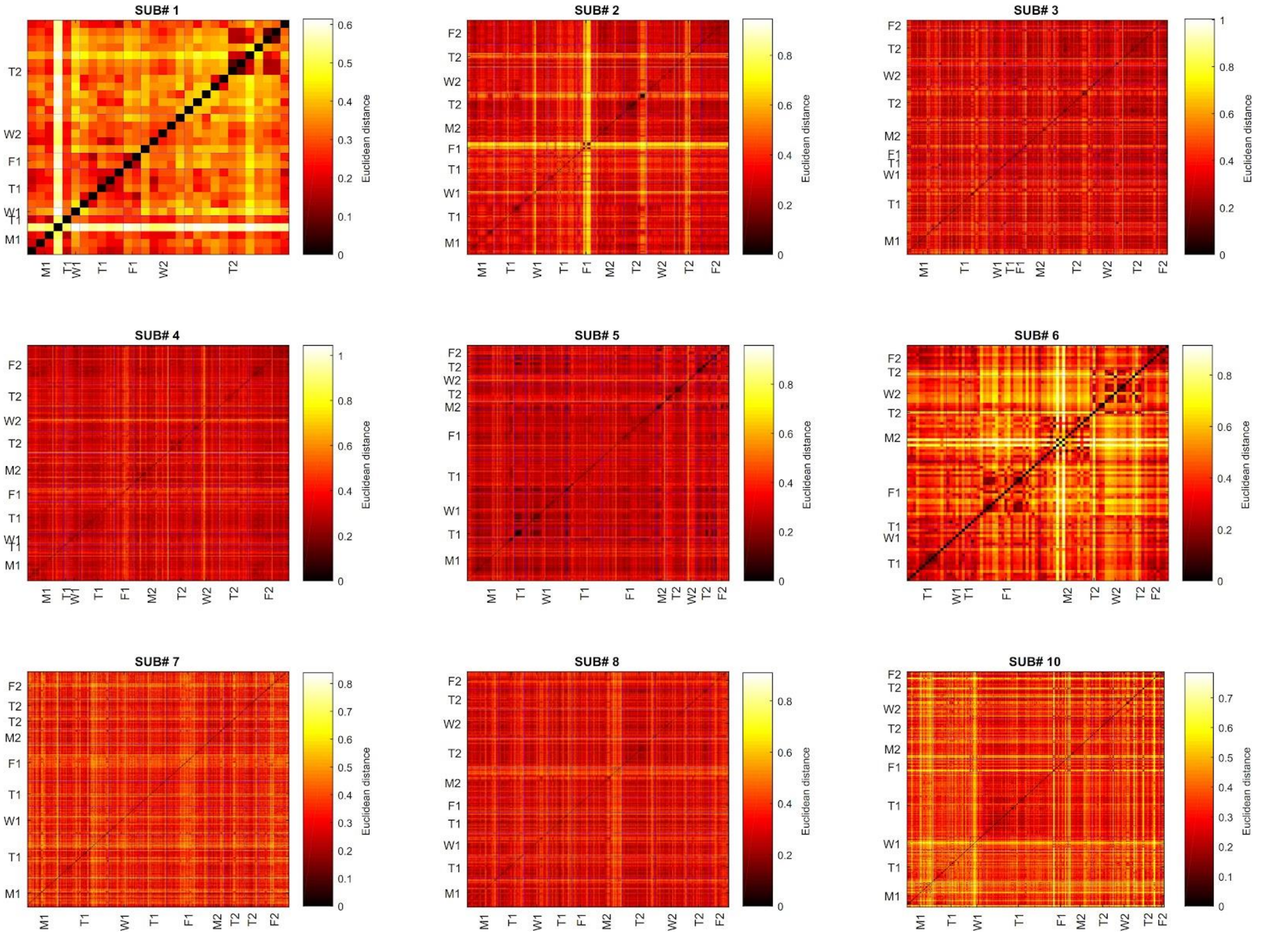

Figure S1-1. Individual recurrence plots. Subject 1 to 10 (subject 9's plot is presented in the main text).

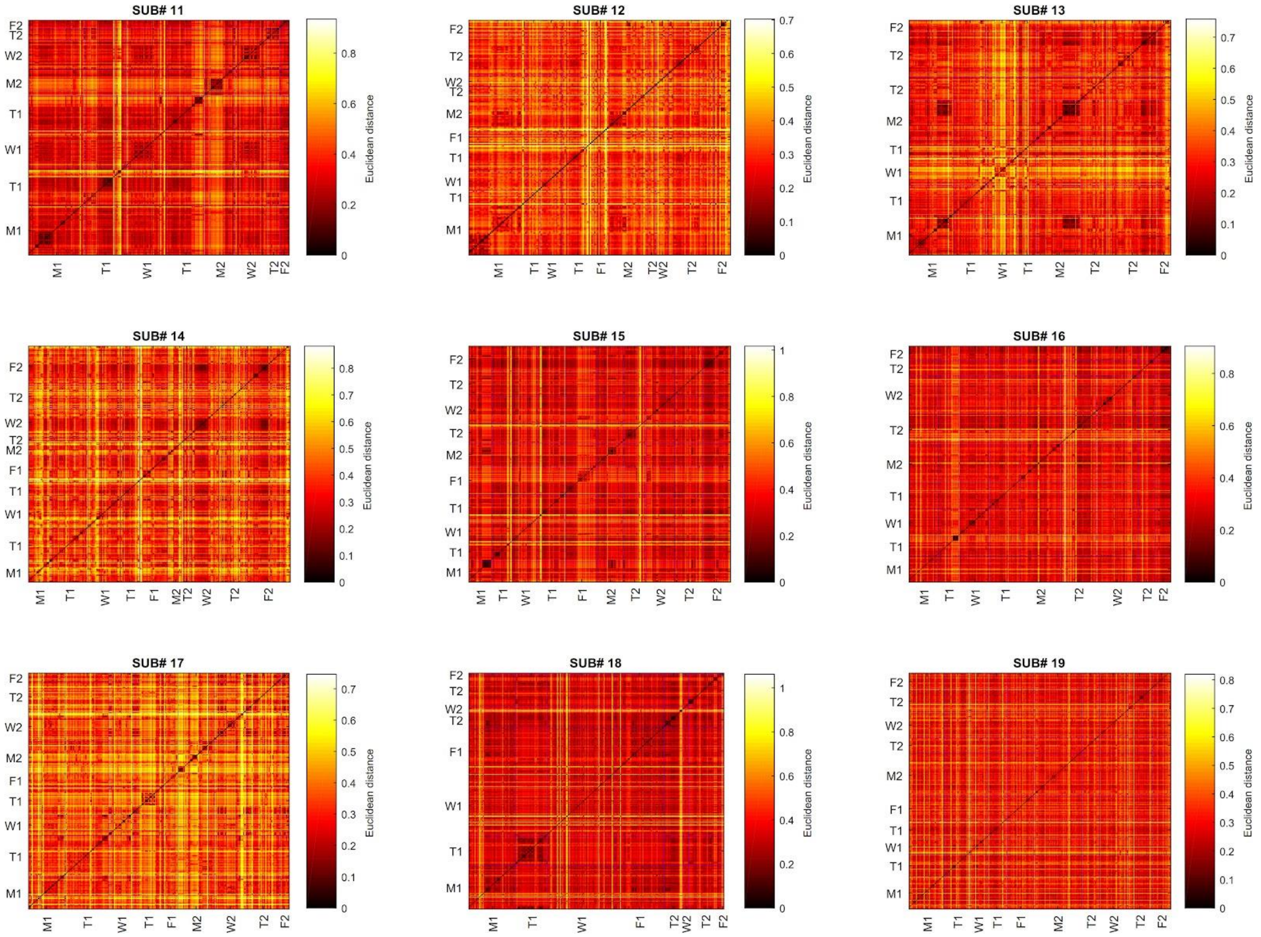

Figure S1-2. Individual recurrence plots. Subject 11 to 19.

Formal evaluation of the recurrence plots.

We used Determinism, Average Diagonal Length, and Divergence measure to evaluate the recurrence in the recurrence plots (Webber & Zbilut, 1994; Zbilut & Webber, 1992). The data was first converted to binary values by thresholding on the median for each participant. Then empirical sampling distributions were generated by permuting the values in the recurrence matrices and taking means for 1000 times. The reported values are means across participants, and the p-values provided are empirical p-values ($p_{empirical}$).

1. Determinism (DET)
   - Determinism measures the proportion of diagonal data points (points forming a diagonal shape, which is an indication of recurrence) among the total data points.

$$DET = \frac{\sum_{l=l_{min}}^{N} lP(l)}{\sum_{l=1}^{N} lP(l)}$$

, where $P(l)$ is the frequency distribution of the lengths l of the diagonal lines, and l_{min} was set to 2. Results showed a Determinism of 0.4946 with $p_{empirical} < 0.001$.

2. Average Diagonal Length (L)
   - Average Diagonal Length measures the length of the diagonal patterns shown in the data points.

$$L = \frac{\sum_{l=l_{min}}^{N} lP(l)}{\sum_{l=l_{min}}^{N} P(l)}$$

, where $P(l)$ is the frequency distribution of the lengths l of the diagonal lines, and l_{min} was set to 2. Results showed an Average Diagonal Length of 3.1063 with $p_{empirical} < 0.001$.

3. Divergence (DIV)
   - Divergence is the inverse of the maximal diagonal line

$$DIV = \frac{1}{L_{max}}$$

, where L_{max} is the maximal length of the diagonal line, and l_{min} was set to 2. Results showed a Divergence of 0.0039 with $p_{empirical} < 0.001$.

2. Statistical analyses conducted at the subject level

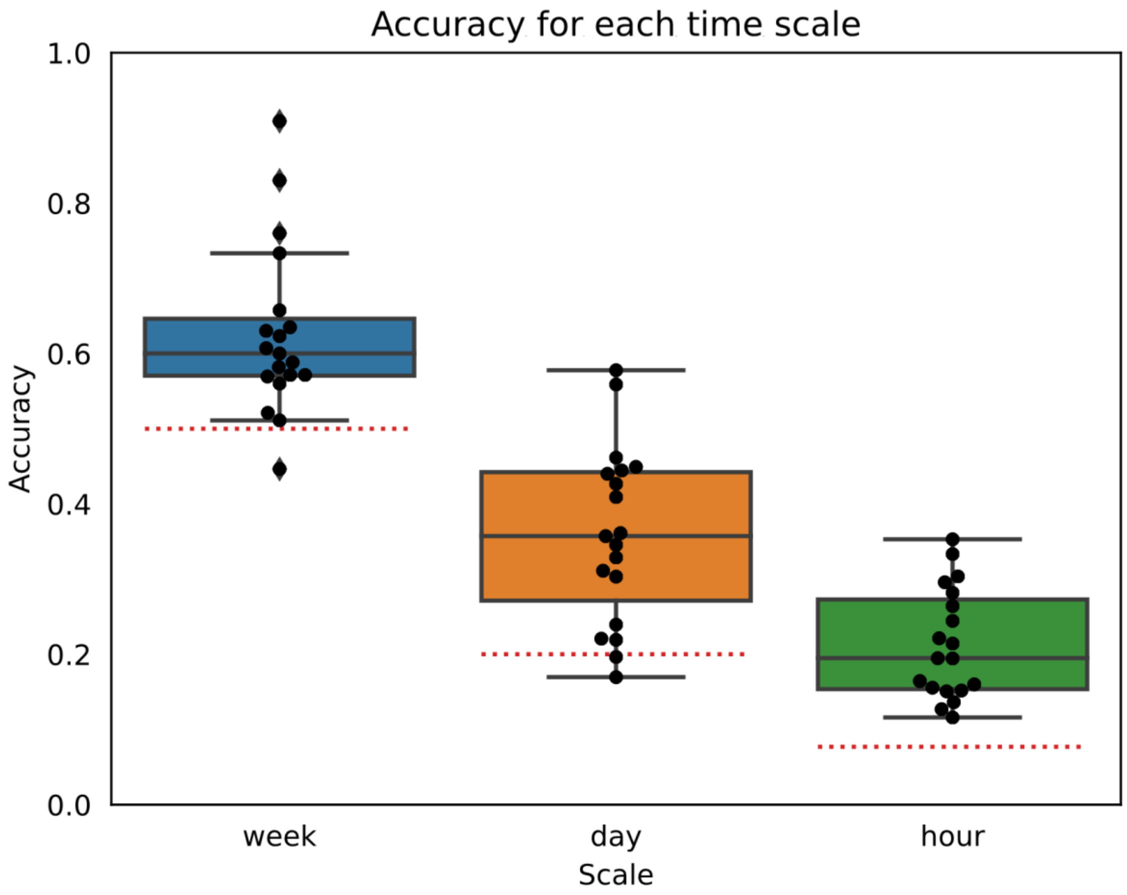

Figure S2-1. Accuracy for each time scale. Red dotted lines represent chance levels (i.e., 1/2 for week, 1/5 for day, and 1/13 for hour). A one-sample t-test against the chance level showed above chance level accuracy for all scales (Week: $M = .63$, $SD = .11$, $p < .001$; Day: $M = .36$, $SD = .11$, $p < .001$; Hour: $M = .21$, $SD = .07$, $p < .001$). p-values where all Holm-Bonferroni corrected (HBC) corrected.

Table S2-1

Analysis on accuracy for hours with individual chance-level.

1. Subject number	2. Accuracy	3. Max hour	4. Number of test trials	5. Accuracy Minus chance-level
1	0.35294	9	34	0.24183
2	0.33333	14	75	0.26190
3	0.30357	13	56	0.22665
4	0.28182	13	110	0.20489
5	0.26415	15	53	0.19748
6	0.24444	10	90	0.14444
7	0.22131	15	122	0.15464
8	0.21429	12	42	0.13095
9	0.19718	11	71	0.10627
10	0.19481	9	77	0.08369
11	0.19444	12	72	0.11111
12	0.16438	17	73	0.10556
13	0.16000	14	25	0.08857
14	0.15556	12	45	0.07222
15	0.15179	17	112	0.09296
16	0.15069	11	73	0.05978
17	0.13636	9	22	0.02525
18	0.12698	14	63	0.05556
19	0.11594	21	69	0.06832

- Each individual was examined using their own chance-level (3rd column), then we subtract each individual's chance level from each individual's accuracy generating a difference from chance score (5th column). Using this score, we can conduct a one-sample t-test against zero, which we see a statistically significant difference above zero (t = 8.19, p < .001).

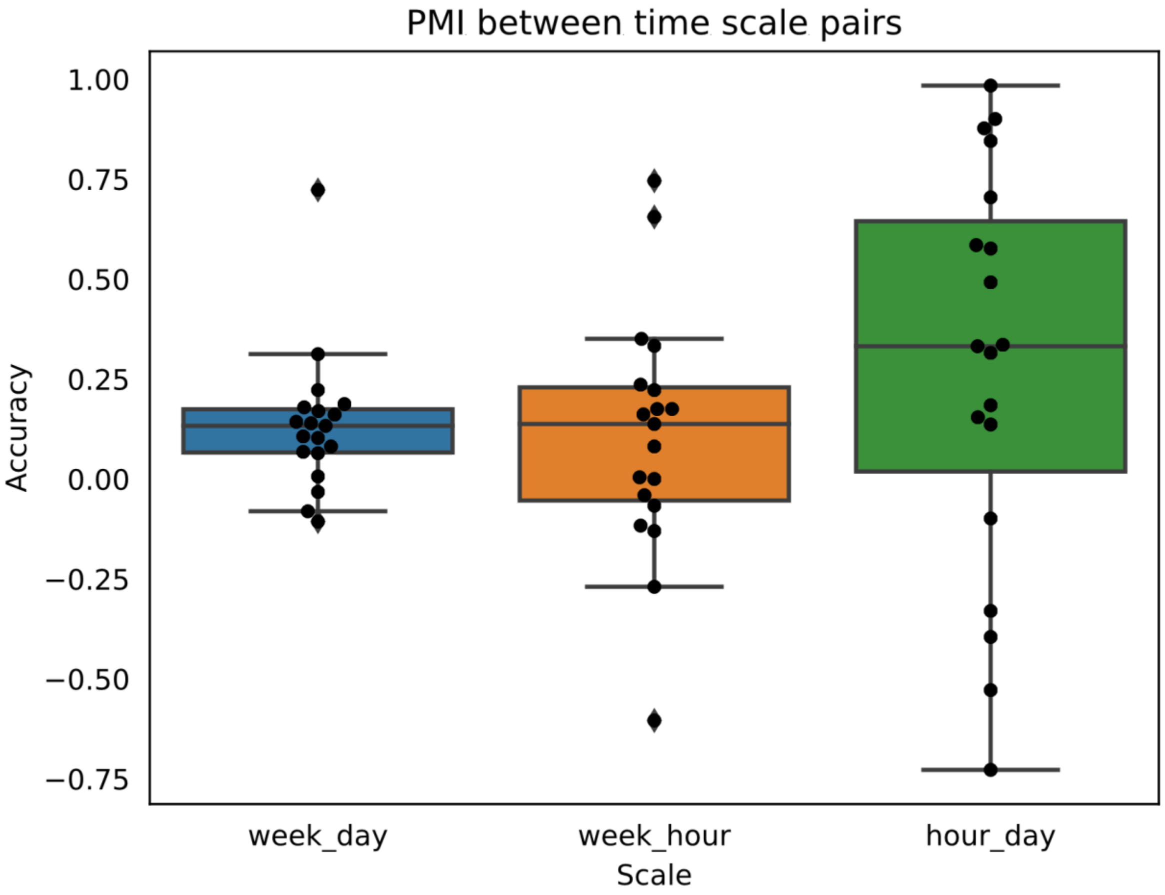

Figure S2-2. PMI between time scale pairs. A one-sample t-test against 0 showed a statistically significant result or a tendency above 0 (PMI(Week, Day): M = .14, SD = .17, p = .003; PMI(Week, Hour): M = .11, SD = .30, p =.17 ; PMI(Hour, Day): M = .28, SD = .49, p = .03). p-values were all Holm-Bonferroni corrected (HBC) corrected.

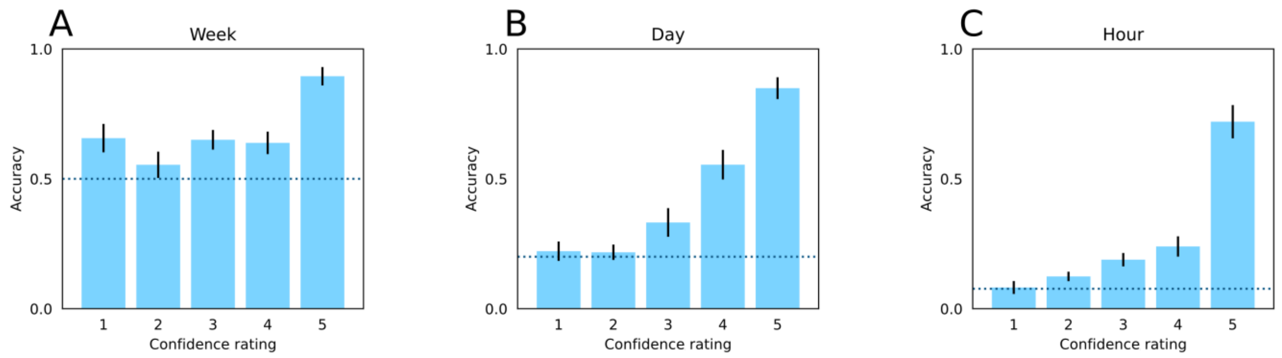

Figure S2-3. Accuracy by confidence rating for (A) week, (B) day, and (C) hour. Values on the x-axis represent confidence rating scores from 'Not at all confident' (1) to 'Very confident' (5). Dotted lines represent chance level for each time scale, error bars represent the standard error of mean. A one-way ANOVA showed statistical significant results for all scales **(**Week: $F(4, 83) = 7.24$, $p < .001$; Day: $F(4, 86) = 32.17$, $p < .001$; Hour: $F(4, 84) = 48.13$, $p < .001$). Error bars represent ± 1 standard deviation of mean.
